# Clinical and Biochemical Phenotype Across the Genotypic Spectrum of 21-hydroxylase Deficiency in 457 Individuals

**DOI:** 10.1210/clinem/dgaf546

**Published:** 2025-10-06

**Authors:** Qizong Lao, Annie Schulman, Sarah Kulkarni, Sarah Kollender, Daniella Bick, Amy Moon, Deepika Burkardt, Deborah P Merke

**Affiliations:** Department of Pediatrics, National Institutes of Health Clinical Center, Bethesda, MD 20892, USA; Department of Pediatrics, National Institutes of Health Clinical Center, Bethesda, MD 20892, USA; Department of Pediatrics, National Institutes of Health Clinical Center, Bethesda, MD 20892, USA; Department of Pediatrics, National Institutes of Health Clinical Center, Bethesda, MD 20892, USA; Department of Pediatrics, National Institutes of Health Clinical Center, Bethesda, MD 20892, USA; Department of Pediatrics, National Institutes of Health Clinical Center, Bethesda, MD 20892, USA; Department of Pediatrics, National Institutes of Health Clinical Center, Bethesda, MD 20892, USA; Division of Genetics and Metabolism, Children's National Hospital, Washington, DC 20010, USA; Department of Pediatrics and Orthopedics, George Washington University, Washington, DC 20052, USA; Department of Pediatrics, National Institutes of Health Clinical Center, Bethesda, MD 20892, USA; *Eunice Kennedy Shriver* National Institute of Child Health and Human Development, Bethesda, MD 20892, USA

**Keywords:** congenital adrenal hyperplasia, 21-hydroxylase deficiency, CAH, CAH-X, *CYP21A2*, genotype-phenotype

## Abstract

**Context:**

Genetic testing for 21-hydroxylase deficiency (21OHD) is advantageous when hormonal testing is equivocal, to molecularly confirm diagnosis, and for genetic counseling.

**Objective:**

To characterize the clinical and biochemical phenotype across the genotypic spectrum of 21OHD in a large cohort using updated genetic methodology.

**Design:**

Retrospective study of 457 individuals with 21OHD enrolled in a Natural History Study at the National Institutes of Health Clinical Center.

**Results:**

The majority (79%) were compound heterozygous, 46% with chimeric alleles/30-kb deletions including 2.6% with attenuated chimeras, 10.1% with CAH-X (33% with cardiac defects), and 3.7% with genotype-phenotype discordance. The most common mutations among individuals with salt-wasting, simple-virilizing, and nonclassic (NC) phenotypes were In2G, I172N, and V281L, respectively. Rare or novel mutations accounted for 4.3% alleles, 0.33% arose *de novo*. 17OHP levels at diagnosis varied by genotype group (Null > In2G > simple-virilizing genotypes > P30L > Other NC; *P* < .001); but maximum values obtained during clinical care over time were similar among all classic and among all NC genotypes. Individuals with P30L had higher 17OHP and lower cortisol at diagnosis compared to other NC genotypes (*P* < .001) and were more likely to have basal 17OHP >1000 ng/dL (*P* < .001). Individuals with cryptic NC CAH had lower 17OHP after cosyntropin stimulation compared to those with symptomatic NC CAH (*P* = .02).

**Conclusion:**

A continuum of disease phenotypes exists with biochemical overlap that increases with age. Improving genotype accuracy to include chimera subtyping to identify attenuated chimeras and CAH-X and consideration of P30L as a unique group are important to guide genetic counseling and provide anticipatory guidance in disease management.

The most common type of congenital adrenal hyperplasia (CAH), an autosomal recessive inborn error of steroidogenesis, is due to mutations in *CYP21A2*, the gene encoding 21-hydroxylase (21OH). Biochemically, 21-hydroxylase deficiency (21OHD) results in decreased conversion of 17-hydroxyprogesterone (17OHP) and progesterone to 11-deoxycortisol and 11-deoxycorticosterone, respectively, with subsequent decreased cortisol and aldosterone synthesis. Loss of cortisol negative feedback on the pituitary leads to increased ACTH-driven production of adrenal-derived androgens (1). The diagnosis is based on elevated 17OHP levels (2).

21OHD CAH falls along a spectrum of clinical presentations (3). Classic 21OHD, with an incidence of approximately 1:15 000, is defined by cortisol deficiency, androgen excess, and varying degree of aldosterone and epinephrine deficiency, whereas the more common nonclassic (NC) form (incidence 1:200-1000) is associated with mostly androgen excess (4, 5). Salt-wasting adrenal crisis in the early neonatal period and 46,XX atypical genitalia are the hallmarks of classic salt-wasting (SW) CAH characterized enzymatically with minimal or absent (<1%) 21OH activity (3). Along the spectrum, individuals who maintain up to 5% of 21OH activity retain the ability to synthesize low amounts of aldosterone (classic simple virilizing [SV] form), presenting with clinical signs of androgen excess including atypical genitalia in 46,XX infants, and signs of precocious adrenarche/puberty such as pubic hair and growth acceleration in toddlerhood if not detected by neonatal screening (5). Individuals with the NC form retain higher levels of enzyme activity, 20% to 50%, and may have a wide range of symptoms including early puberty or early pubic hair in both sexes, hirsutism, irregular menses or infertility in females, or may be clinically asymptomatic and discovered through genetic testing of family members of individuals with CAH (“cryptic” CAH) (6). Biochemically, about one-third of individuals with NC CAH have suboptimal cortisol response (7, 8). Historically, levels of 17OHP have been used to help distinguish phenotype and genotype subtypes, with more severe mutations corresponding to higher 17OHP levels both basal and 60 minutes following cosyntropin stimulation (9, 10).

*CYP21A2* is mapped to the *RCCX* module in the major histocompatibility complex of chromosome 6 (6p21.33). This locus is a typical low-copy repeat featuring highly homologous gene pairs with commonly existing copy number variation (11, 12). The term “*RCCX”* stands for “*RP-C4-CYP21-TNX*” in tandem: “*RP*” signifies *STK19* encoding serine/threonine kinase 19 and pseudogene *STK19P* (formally named *RP1* and *RP2,* respectively); “*C4*” signifies *C4A* and *C4B* encoding 2 isotopes of complement component 4; “*CYP21*” signifies *CYP21A2* and pseudogene *CYP21A1P*; and “*TNX*” signifies *TNXB* encoding tenascin-X, a glycoprotein crucial for the extracellular matrix composition, and pseudogene *TNXA.* The “repeat” involves a unit of *C4-CYP21A1P-TNXA-STK19P*, whose copy number variations are in general responsible for the local structural complexity (11).

The entire *RCCX* locus is error prone during meiosis, which is in fact the main mechanism underlying *CYP21A2* defects. Among the 10 most common pathogenic variants that account for 60% of the total 21OHD alleles, 9 are minor conversions termed for the misalignments of deleterious *CYP21A1P* fragments into *CYP21A2*; approximately 30% of the 21OHD alleles are chimeric genes, and major conversions in some cases, both involving chunk misalignments of pseudogene sequence in multiple kb scale (13-16). The chimeric genes, often termed as 30-kb deletions, can be further categorized into 3 subgroups: “attenuated” CAH (*CYP21A1P/CYP21A2*) chimeras that only impair the affected allele moderately (17-19); “classic” CAH chimeras that nullify the affected allele (18); and “CAH-X” (*CYP21A1P-TNXA/TNXB*) chimeras that not only nullify the affected *CYP21A2* allele but also impair the neighboring *TNXB*, which leads to a contiguous syndrome of CAH-X, a combination of autosomal recessive CAH plus autosomal dominant hypermobility Ehlers-Danlos syndrome (hEDS) (20-24). CAH-X chimeras have been estimated to account for approximately 30% of all 30-kb deletions or 10% to 15% of all 21OHD alleles (25-27). There are also more than 200 types of rare pathogenic variants throughout *CYP21A2*, which combine to account for approximately 10% of 21OHD allele frequency, but do not share homology with *CYP21A1P* (13, 16). Most affected 21OHD individuals are compound heterozygotes with different *CYP21A2* variants in each allele (1, 14, 15). The less severely impaired allele correlates to the residual 21OH activity, which in turn determines the disease severity, or the CAH phenotype.

In 2011, we reported a summary of 21OHD genotype-phenotype correlations based on 182 unrelated families followed in a Natural History Study at the National Institutes of Health (NIH) Clinical Center (14). Here we provide an updated report of our large cohort, now composed of 457 individuals with 21OHD CAH from 381 unrelated families. We have since standardized our genetic testing with a Sanger methodology that covers both *CYP21A2* and *TNXB* exons 32 through 44 in base-pair resolution, which offered the most complete and comprehensive scope of 21OHD genetics available. We have clarified many previous discrepancies resulting from the limitations of prior genetic testing methodologies. We have classified the 3 chimeric gene subgroups (attenuated CAH, classic CAH, and CAH-X) of the 30-kb deletion alleles, which were in general reported as classic *CYP21A2* null variants previously. We have also identified several novel variants. Furthermore, we provide correlations of genotype groups to clinical and biochemical phenotype, including the spectrum of 17OHP at diagnosis and during therapy. We believe this report will significantly enhance our understanding of 21OHD CAH genetics and genotype-phenotype associations.

## Methods

### Study Design and Participants

Individuals were enrolled in a Natural History Study at the NIH Clinical Center (Bethesda, MD) (www.ClinicalTrials.gov; Identifier no. NCT00250159) and were recruited through self-referral and/or physician referral through listings on www.Clinicaltrials.gov. The diagnosis of CAH due to 21OHD was confirmed by biochemical and genetic testing.

Phenotypic classification was determined by 1 pediatric endocrinologist (D.P.M.) based on clinical and hormonal criteria and a retrospective review of each individual's medical records as previously described (14). The study was approved by the NIH Institutional Review Board. All adults and a parent or guardian of children younger than age 18 years provided written consent. Assent was obtained in children ages 7 years and older. This report is based on genetic and clinical information obtained through June 30, 2025.

### Molecular Genetic Analysis

In our Natural History Study, genotyping of families was performed by multiplex minisequencing and conversion-specific PCR assay (MMCP) (Esoterix Laboratory Services, Inc, Calabasas Hills, CA). Starting in 2016, comprehensive Sanger sequencing analysis with the classic CYP779f/Tena32f allele-specific long PCR (PreventionGenetics, LLC, Marshfield, WI) was also performed. All families enrolled before 2016 were also subject to genotyping by Sanger analysis if gDNA samples were available. For each family, their complete genotype results, including the haplotypes of compound heterozygous variants, were determined by family-wide genetic analysis, if available. Overall, family studies were performed for 72% of the cohort (302 with parental gDNA, 27 with offspring gDNA). Chimeric gene subtypes were determined by an array of variants with pseudogene origin as described previously (18). Individuals with milder than expected phenotypes or duplication suggested by other genetic testing results (eg, MMCP) were subject to *CYP21A2* duplication testing (28).

The references for genetic analysis were *CYP21A2* (NM_000500.7) and *TNXB* (NM_019105.6). Pathogenic variants were presented in traditional common names or following amino acid nomenclature rules to maintain continuity with historical milestone literature and clinical reports (1, 15, 29, 30).

### Genotype Groups

21OHD genotypes were grouped based on residual 21OH activity as previously described (14, 29-31). Group 0 consisted of genotypes of biallelic null variants that totally abolish 21OH activity and were expected to be associated with an SW phenotype. Typical variants in group 0 included large deletions (classic CAH chimeras, CAH-X chimeras, large conversion), G110Efs, E6 cluster, L307fs, Q318X, and R356W. Group A genotypes, mostly defined by the In2G variant, were expected to have an SW phenotype in general, as this mutation is associated with minimal <1% residual enzymatic activity; however, an SV phenotype has been well described with a prevalence of 8% to 20% (14, 15). As a result, we did not consider SV phenotype in group A to be discordant. Group B genotypes, associated with 1% to 5% of enzyme activity, were expected to have an SV phenotype, with the most common defining variants I172N and attenuated CAH chimeras featuring a P30L variant *in cis* with a *CYP21A1P* promoter. Group BC genotype was newly created in this study for genotypes defined by the variant P30L. Although P30L has been traditionally considered an NC variant, phenotypes often vary between SV and NC (15, 30, 32, 33). Group C genotypes, estimated to have 20% to 50% residual 21OH activity, were expected to be associated with an NC phenotype. Their typical defining variants include V281L, P453S, and untranslated region variants. Group D comprised unidentifiable/indeterminant genotypes or phenotypes.

### Biochemical Assays

Morning fasting blood was collected before taking medications and was analyzed at the NIH Clinical Center Laboratory, unless otherwise specified. Serum 17OHP levels were measured by liquid chromatography-tandem mass spectrometry (Mayo Medical Labs) and cortisol serum levels were measured by chemiluminescence immunoassay (NIH).

### Clinical and Biochemical Data

NIH medical records and, if available, outside medical records were reviewed retrospectively to compile clinical and biochemical data. Age of diagnosis was determined based on review of initial endocrine evaluation and date of medication initiation, assigned on a case-by-case basis and reviewed by 1 pediatric endocrinologist (D.P.M.).

Hormonal data were retrospectively collected for 3 purposes. First, serum levels of 17OHP at initial diagnosis of 21OHD were recorded (n = 199) to evaluate if the level of 17OHP at diagnosis reflected the traditional estimated degree of enzyme impairment. Second, individuals’ highest 17OHP serum level documented in medical records was obtained (n = 405) to evaluate maximum 17OHP in relation to genotype group. Thirty-six (11.2%) individuals with classic CAH were excluded from the biochemical-genotype analysis because all available 17OHP levels were suppressed due to supraphysiologic glucocorticoid therapy. Third, 17OHP serum levels were collected for individuals with NC CAH who underwent cosyntropin stimulation testing to create an updated reference nomogram. Overall, 63 (46.7%) of individuals with NC CAH were treated with glucocorticoid therapy at some time during their lives; cosyntropin stimulation data presented are around the time of diagnosis and before receiving glucocorticoid therapy. Untreated individuals with NC CAH underwent a cosyntropin stimulation test at NIH (n = 48) or at a local health care facility (n = 41). Cortisol and 17OHP levels at baseline and 60 minutes after stimulation were collected. Nomograms of serum 17OHP and cortisol were constructed to evaluate the P30L mutation in contrast to other NC-associated mutations.

### Statistical Analysis

Statistical analysis was performed using R v.4.4.1 (R Foundation for Statistical Computing, Vienna, Austria). Summary statistics (mean ± SD or median [IQR]) were calculated. 17OHP values at diagnosis were log-transformed to normalize the data. Student *t*-tests, Welch *t*-tests, Wilcoxon rank-sum tests, ANOVA, and Kruskal-Wallis tests were used to assess differences in 17OHP and cortisol by genotype group or phenotype as per data distributional assumptions. Pairwise comparisons were performed using Scheffe post hoc tests or Bonferroni correction for parametric and nonparametric tests, respectively. An additional contrast test was run using the Scheffe method to assess the difference in 17OHP values at diagnosis between genotype groups 0/A and B. A *P* <.05 was considered significant.

## Results

### Cohort

Our large Natural History Study cohort of 21OHD CAH was composed of 457 individuals from 381 unrelated families (262 females, 195 males). Their self-reported race was: 85.3% White, 2.6% African American or Black, 3.1% Asian, 0.2% Native American or Native Alaskan, 2.8% multiracial, and 6% unknown. Ethnically, 7.9% identified as Hispanic or Latinx, whereas 92.1% reported as non-Hispanic, non-Latinx, or unknown ethnicity. A total of 322 individuals had classic CAH phenotypes (211 SW, 111 SV). Of the 135 individuals with NC CAH, 111 (82.2%) were diagnosed based on CAH-related symptomatology, whereas 24 (17.8%) individuals had cryptic CAH diagnosed based on family genetic studies, with the diagnosis confirmed by hormonal testing. Overall, 5.3% (24/457) of parents had cryptic CAH.

Individuals were classified into the 6 genotype groups based on their expected phenotype-defining (ie, milder gene defect) allele: Group 0 (n = 86); Group A (n = 130); Group B (n = 100); Group BC (n = 16); Group C (n = 122), and Group D (n = 3) (Table 1).

**Table 1.** Genotypes and phenotypes of 457 individuals with congenital adrenal hyperplasia due to 21-hydroxylase deficiency

Genotype category	Allele 1	Allele 2	Allele 3	Phenotype		
				SW	SV	NC (cryptic)
Group 0	CAH CH-1	CAH CH-1		3		
	CAH CH-1	CAH CH-3		1		
	CAH CH-1	CAH CH-5		5		
	CAH CH-1	CAH CH-5*^a^*		1		
	CAH CH-1	CAH CH-5*^b^*		1		
	CAH CH-1	CAH CH-8		2		
	CAH CH-1	G110Efs		3		
	CAH CH-1	G110Efs, V281L		1		
	CAH CH-1	H365Y		1		
	CAH CH-1	IVS8 + 1G > A		1		
	CAH CH-1	large conversion*^c^*, H62L		1		
	CAH CH-1	Q318X		2		
	CAH CH-1	R226fs		1		
	CAH CH-1	R356W		1		
	CAH CH-1	R426C		1		
	CAH CH-3	CAH CH-3		1		
	CAH CH-3	Q318X		1		
	CAH CH-5	CAH CH-5		12		
	CAH CH-5*^a^*	CAH CH-5*^a^*		1		
	CAH CH-5	CAH CH-8		1		
	CAH CH-5	G110Efs		1		
	CAH CH-5	H365Y		1		
	CAH CH-5*^b^*	In2G, G110Efs		1		
	CAH CH-5	IVS8 + 1G > A		1		
	CAH CH-5	Q318X		2		
	CAH CH-5*^a^*	Q318X		1		
	CAH CH-5	R356W		3		
	CAH CH-5*^b^*	R356W		2		
	CAH CH-5	R444X		1		
	CAH CH-5	R483fs		1		
	CAH CH-6	CAH CH-6		1		
	CAH CH-8	G110Efs, V281L		1		
	CAH-X CH-1	CAH CH-1		1		
	CAH-X CH-1	CAH CH-3		1		
	CAH-X CH-1	CAH CH-5		2		
	CAH-X CH-1	CAH-X CH-2		1		
	CAH-X CH-1	large conversion*^d^*		4		
	CAH-X CH-1	L307fs		1		
	CAH-X CH-2	CAH CH-1		1		
	CAH-X CH-2	CAH CH-5		2		
	CAH-X CH-2	CAH-X CH-2		1		
	CAH-X CH-2	CAH-X CH-3		1		
	CAH-X CH-2	In2G, R483fs		2		
	CAH-X CH-2	R483fs		1		
	G110Efs	Q318X, R356W		1		
	G110Efs	R356W		1		
	large conversion*^e^*, H62L	large conversion*^f^*		1		
	Q318X	E6 cluster		1		
	Q318X, L307fs	E6 cluster		1		
	Q318X, L307fs	I172N, E6 cluster		1		
	Q318X (*TNXB* c.12463 + 2T > C)	CAH CH-3		2		
	Q318X (*TNXB* c.12463 + 2T > C)	CAH CH-5		1		
	R356W	V281L, G110Efs		2		
Group A	In2G	30-kb deletion*^g^*		1		
	In2G	C423fs		1		
	In2G	CAH CH-1		13		
	In2G	CAH CH-1	Q318X	1		
	In2G	CAH CH-2		1		
	In2G	CAH CH-3		1		
	In2G	CAH CH-5		17		
	In2G	CAH CH-5*^a^*		2		
	In2G	CAH CH-5*^b^*		2		
	In2G	CAH CH-8		2	2	
	In2G	CAH-X CH-1		7		
	In2G	CAH-X CH-2			1	
	In2G	E247fs		1		
	In2G	E6 cluster		1		
	In2G	E6 cluster, V281L		1		
	In2G	G110Efs		3		
	In2G	G484fs			1	
	In2G	H365Y		3		
	In2G	In2G		21	6	
	In2G	In2G, P453S		3		
	In2G	In2G, V281L		2		
	In2G	IVS9-1G > A		1		
	In2G	L307fs		2		
	In2G	large conversion*^c^*		1		
	In2G	large conversion*^h^*		1		
	In2G	Q262fs		1		
	In2G	Q318X		5		
	In2G	R354C		1		
	In2G	R356W		5	2	
	In2G	R408H			2	
	In2G	S170fs		1		
	In2G	W405X		2		
	In2G, V281L	30-kb deletion*^g^*		1		
	In2G, V281L	CAH CH-1		1		
	In2G, V281L	CAH-X CH-1		1		
	In2G, V281L	CAH-X CH-2		3		
	In2G, V281L	Q318X		1		
	In2G, V281L	R356W		1		
	In2G, P453S	CAH CH-5		1		
	In2G, P453S	CAH-X CH-1		1		
	In2G, P453S	Q318X		2		
	In2G, P453S	Q318X	In2G	1		
Group B	CAH CH-4	CAH CH-1			1	
	CAH CH-4	CAH CH-4/9*^i^*				1
	CAH CH-4	CAH CH-5			1	
	CAH CH-4	In2G			1	
	CAH CH-4/9*^i^*	CAH CH-5*^a^*			1	
	CAH CH-4/9*^i^*	I172N			1	
	CAH CH-4/9*^i^*	In2G			2	
	CAH CH-4/9*^i^*	Q318X			1	
	CAH CH-10	CAH CH-1*^k^*			1	
	G424S	In2G			1	
	I172N	C168X			1	
	I172N	CAH CH-1		3	12	
	I172N	CAH CH-1	large conversion*^d^*		1	
	I172N	CAH CH-1	large conversion*^l^*		1	
	I172N	CAH CH-1*^k^*, H62L			1	
	I172N	CAH CH-5		1	5	
	I172N	CAH CH-5*^b^*			4	
	I172N	CAH-X CH-1			3	
	I172N	CAH-X CH-2			1	
	I172N	E6 cluster			1	
	I172N	G110Efs			1	
	I172N	I172N		1	6	1
	I172N	I172N (*TNXB* p.I2302fs)			1	
	I172N	In2G		1	20	
	I172N	In2G, R483fs			1	
	I172N	In2G, V281L			2	
	I172N	In2G	large conversion*^m^*		1	
	I172N	L307fs			2	
	I172N	P30fs			1	
	I172N	Q318X			5	
	I172N	R356W		1	4	
	I172N	R483fs			1	
	I172N	V281L, L307fs			1	
	I172N	W405X		1		
	I172N, A265V	Q318X		1		
	I172N, P453S	CAH-X CH-1			1	
	I77T	CAH-X CH-2			1	
	P463L	Q318X			1	
Group BC	P30L	CAH CH-1				1
	P30L	CAH CH-3			1	
	P30L	CAH-X CH-2				1
	P30L	E351K				1
	P30L	I172N				5
	P30L	In2G			1	3
	P30L	In2G, P453S				2
	P30L	Q318X	In2G			1 (1)
Group C	c.-113G > A, c.-110T > C, c.-103A > G	CAH CH-1				1 (1)
	c.*13G > A	CAH CH-1				1
	c.*13G > A	P453S				1 (1)
	H120R	I172N				1
	P453S	CAH CH-1				1 (1)
	P453S	CAH CH-8				1 (1)
	P453S	CAH-X CH-2				1 (1)
	P453S	I172N				3 (2)
	P453S	In2G				2 (1)
	P453S	large conversion*^h^*				1
	P482S	I172N				1 (1)
	R124C	In2G				1
	R124C	R124C				1
	V281L	30-kb deletion*^g^*				1 (1)
	V281L	CAH CH-1				10
	V281L	CAH CH-1*^k^*, H62L				1
	V281L	CAH CH-5				3 (1)
	V281L	CAH CH-5*^b^*				1
	V281L	CAH CH-8				1
	V281L	CAH CH-9				2
	V281L	CAH-X CH-1				1
	V281L	CAH-X CH-2				2
	V281L	G110Efs				2
	V281L	I172N				6 (2)
	V281L	I172N, V281L			1	
	V281L	I77T				1
	V281L	In2G			3	13 (3)
	V281L	In2G, V281L				2 (1)
	V281L	M284V				1
	V281L	P453S				4
	V281L	P482S				1 (1)
	V281L	Q318X				5 (1)
	V281L	R356W				2
	V281L	R426P				1
	V281L	V237E				1
	V281L	V281L				40 (3)
	V281L	V281L or 30-kb deletion*^g^*				1 (1)
Group D	30-kb deletion*^g^*	R356W			1	
	In2G	Y98D				1
	In2G, G110Efs, I172N (phase unknown)				1	
			Total	211	111	135 (24)

NC CAH is presented in both total and (NC-cryptic) case counts.

^*a*^CAH CH-5 without V281L.

^*b*^CAH CH-5 w/o V281L, with alternative E6 cluster: c.710T > A, c.711C > G.

^*c*^large conversion: P30L-In2G- G110Efs.

^*d*^large conversion: I172N-E6 cluster-V281L-L307fs.

^*e*^large conversion: P30L-In2G-G110Efs-I172N-E6 cluster-L307fs.

^*f*^large conversion: In2G-G110Efs-I172N-E6 cluster-V281L-L307fs-Q318X-R483fs.

^*g*^unspecified chimeras, also collectively termed as 30-kb deletion.

^*h*^large conversion: In2G-G110Efs-I172N-E6cluster.

^*i*^attenuated chimera, subtype unspecified.

^*k*^CAH CH-1 without P30L.

^*l*^large conversion: In2G-G110Efs-I172N-E6 cluster-L307fs-Q318X.

^*m*^large conversion: E6cluster-V281L-L307fs.

### Pathogenic *CYP21A2* Alleles

**Figure 1.**
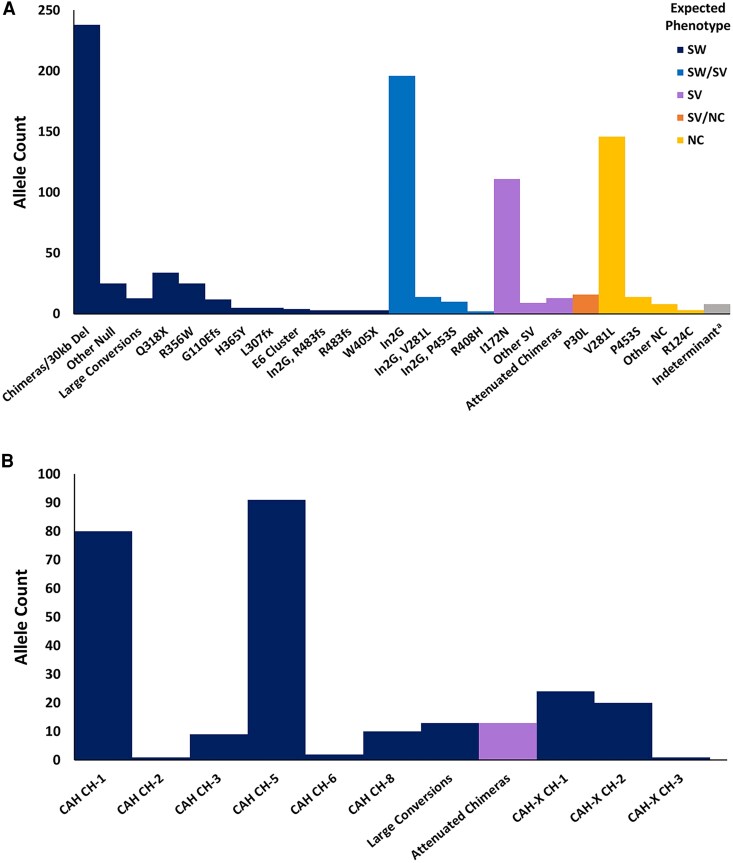
Distribution of *CYP21A2* pathogenic variants. (A) Pathogenic allele counts in a cohort of 457 individuals with congenital adrenal hyperplasia resulting from 21-hydroxylase deficiency, with expected phenotypes shown. (B) Subtypes of the 30-kb deletions/chimeras identified (n = 264 alleles). ^a^Includes undefined 30-kb deletions (n = 4), V28L/V281L or undefined 30-kb deletion (n = 1), In2G/G110Efs/I172N (phase unknown, n = 2), and Y98D (n = 1).

A broad spectrum of pathogenic *CYP21A2* variants was found within our cohort (Fig. 1A). Overall, we identified 920 pathogenic *CYP21A2* or CAH alleles from the 457 affected individuals (Table 1); 6 extra copies were due to *CYPA21A2* duplications. Most CAH alleles were inherited except for 3 alleles (0.33%), 1 each of CAH-X CH-1, Q318X, and I172N, were due to *de novo* mutations in 3 individuals. As expected, the majority of individuals were compound heterozygotes, whereas 96 (21.0%) individuals had homozygous CAH alleles. Most commonly, individuals were homozygous for V281L (n = 40), In2G (n = 27), or CAH CH-5 (n = 12). The majority of individuals (69.6%) with NC CAH were carriers of classic CAH.

The most common CAH alleles were chimeric genes (also known as 30-kb deletions or large conversions), followed by In2G, V281L, and I172N, with allele counts (allele frequency) of 264 (28.7%), 196 (21.3%), 146 (15.9%), and 111 (12.1%), respectively. Among the chimeric genes, classic CAH chimeras that nullify *CYP21A2* were predominant and accounted for 238 alleles (90.2%) of the chimeric genes; the most prevalent subtypes were CAH CH-1 (n = 80) and CAH CH-5 (n = 91) (Fig. 1B). CAH CH-5 had 2 less common subtypes: 1 without the V281L variant (n = 7) and the other having an atypical E6 cluster composed of c.710T > A, c.711C > G variants in addition to missing the V281L variant (n = 11). Attenuated CAH chimeras (CAH CH-4, CAH CH-9, CAH CH-10, or attenuated unspecified), which partially impair *CYP21A2*, accounted for 13 alleles, 4.9% of chimeras, or 1.4% of all CAH alleles. CAH-X chimeras, affecting both *CYP21A2* and neighboring *TNXB,* accounted for 45 alleles in 42 individuals (4.9% of allele frequency; 9.2% of cohort prevalence) including 3 with a biallelic CAH-X chimera genotype. The allele counts of CAH-X CH-1, CH-2, and CH-3 were 24, 20, and 1, respectively. Specification of chimera subtype was not possible for four “30-kb deletion” alleles in 4 individuals from lack of gDNA availability. Thirteen null alleles were determined to be a large conversion because chunk pseudogene sequence was present within the *CYP21A2* gene, whereas the 5′-untranslated region remained intact. This was observed in 12 individuals, 3 of them having *CYP21A2* duplications.

### Genotype-phenotype Correlations

**Figure 2.**
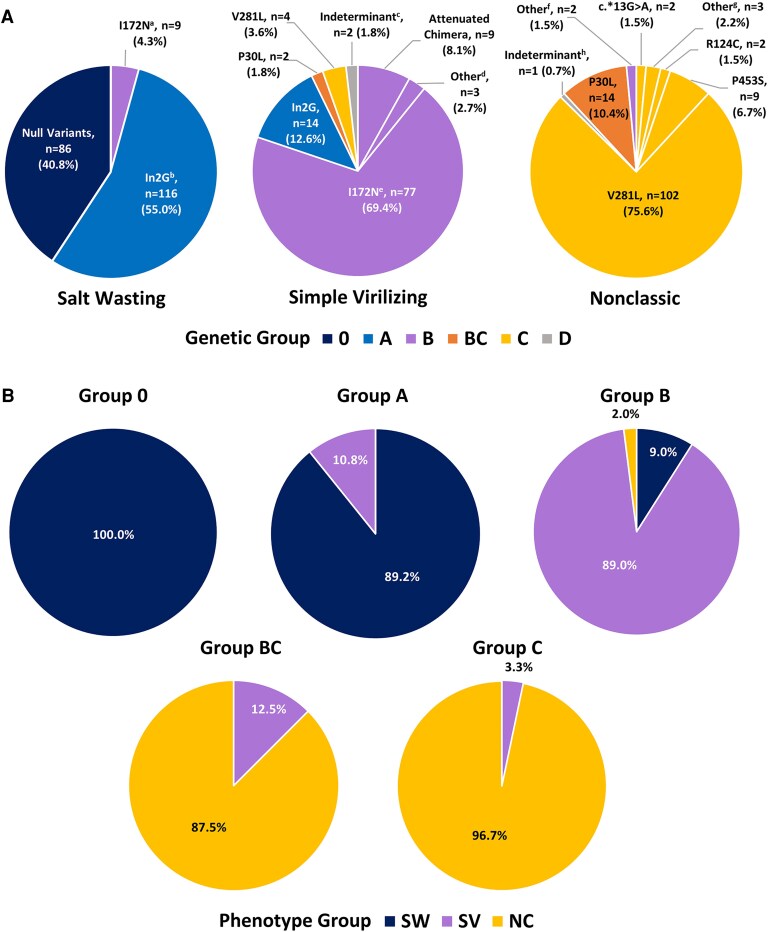
Genotype-phenotype associations in 21-hydroxylase deficiency. (A) Phenotype groups are shown with associated genotypes. Genotypes are listed according to the expected phenotype-defining variant (milder defect) and are color coded according to genotype group. (B) Genotype groups and associated clinically observed phenotypes. ^a^Includes one I172N, A265 V allele. ^b^Includes In2G, P453S (n = 4) and In2G, V281L (n = 8) alleles. ^c^In2G/G110Efs/I172N (phase unknown) and undefined 30-kb deletion. ^d^I77T, P463L, and G242S alleles. ^e^Includes one I172N, P453S allele. ^f^Attenuated chimera, I172N alleles. ^g^H120R, P482S, c.-113G > A/c.-110T > C/c.-103A > G/L199F (VUS) alleles; ^h^Y98D.

Across the phenotypic spectrum, genotype-phenotype discordance was observed in 17 (3.7%) individuals. Among those with an SW phenotype, the majority (95.7%) had either a null or In2G variant (Fig. 2A). Although the majority (69.4%) of individuals with an SV phenotype carried the I172N variant, a variety of pathogenic *CYP21A2* variants also accounted for this milder classic phenotype. The majority (98%) of individuals with NC CAH phenotype carried variants that corresponded to the NC phenotype, with V281L the most prevalent (75.6%).

Groups 0 and A genotypes were highly concordant with their expected phenotypes (Fig. 2B). All 86 individuals with a group 0 null CAH genotype had an SW phenotype; 1 individual with an unspecified “30-kb deletion”/R356W genotype and SV phenotype (diagnosed at 4.5 years with virilization) had no additional gDNA available for further analysis and was therefore considered indeterminant; 116 of 130 (89.2%) individuals with a group A genotype had an SW phenotype, whereas 14 (10.8%) had an SV phenotype. Consistent with prior reports and our own clinical experience, an SV phenotype associated with a group A genotype is expected, occurs in a minority of individuals, and does not represent genotype-phenotype discordance.

Most of the discordance we observed was in the genotype groups associated with moderate to mild CAH severity. Among the 100 individuals with a group B genotype, 89 (89%) had an SV phenotype as expected; 9 had SW with 8 cases having I172N *in trans* with a null allele; 2 had an NC phenotype, 1 with the genotype I172N/I172N and 1 with the genotype of biallelic attenuated CAH chimeras. Nine of 10 individuals with a phenotype-defining attenuated chimera displayed an SV phenotype, as expected. The individual with a biallelic attenuated chimera genotype and a NC phenotype was diagnosed at 9 years of age (bone age 10 years, 6 months) following early development of pubic hair and a positive cosyntropin stimulation test (17OHP: baseline 426 ng/dL, 60 minutes 4117 ng/dL; cortisol: baseline 6.8 μg/dL, 60 minutes 16.5 μg/dL).

The newly created group BC had 16 individuals with genotypes defined by a P30L allele, 14 of which had an NC phenotype, whereas 2 had SV CAH: 1 individual had a positive newborn screen and at 2 months of age had a cosyntropin stimulation test consistent with classic CAH (60 minutes: 17OHP 16 400 ng/dL, cortisol 3.3 μg/dL), and a second individual had significant virilization at 4 years of age with a bone age of 12 years, 6 months. There were 122 individuals carrying a group C genotype, 118 (96.7%) of which had an NC phenotype, as expected. There were 4 cases of SV phenotype in this group, 3 with a genotype of V281L/In2G and 1 with a genotype of V281L/I172N-V281L, including 2 females with SV CAH born with atypical genitalia and diagnosed with CAH within the first week of life, and 2 male siblings with SV CAH diagnosed in early childhood with significant virilization and advanced bone age. Twenty-four individuals had “cryptic” NC CAH (9 males). Males with cryptic CAH were clinically asymptomatic despite their affected genotypes and mild to moderate abnormal hormonal findings, whereas some females had mild symptoms of hyperandrogenism.

### CAH-X

A total of 45 CAH-X chimeric alleles were found in 42 individuals with 21OHD CAH, 3 with biallelic CAH-X. In addition to CAH-X chimeras, there were 2 additional pathogenic *TNXB* variants associated with hEDS: a *TNXB* c.12463 + 2T > C splice site variant as previously described (34) *in cis* with *CYP21A2* Q318X affecting 3 individuals; and a novel *TNXB* c.6904insC (p.I2302fs) variant *in cis* with *CYP21A2* I172N affecting 1 individual. They were termed as “other CAH-X.” The overall CAH-X prevalence in our cohort was 10.1% (46/457).

The CAH phenotype of these individuals with CAH-X was concordant with the CAH genotype in an autosomal recessive manner and was unlikely influenced by the *TNXB* defect. Consistent with its autosomal dominant inheritance, the *TNXB* defects in a monoallelic CAH-X genotype were associated with various degrees of hEDS symptomatology, regardless of the CAH phenotype (Table 2).

**Table 2.** Clinical characteristics of CAH-X individuals by genetic variant

	CAH patients			
	CAH-X CH-1 (n = 21)	CAH-X CH-2 (n = 15)	Biallelic (n = 3)	Splice Site (n = 3)
Age, y	15.7 ± 9.0	19.8 ± 19.6	16.2 ± 10.4	30.0 ± 22.9
Females	11 (52)	10 (67)	0 (0)	2 **(**67)
Musculoskeletal				
Generalized hypermobility*^a^*	10 (56)	8 (53)	3 (100)	1 **(**33)
Small joint hypermobility	13 (72)	12 (80)	3 (100)	2 **(**67)
Large joint hypermobility	7 (39)	9 (60)	3 (100)	1 **(**33)
Subluxations	6 (30)	7 (50)	2 (67)	0 **(**0)
Chronic arthralgia	8 (40)	3 (20)	1 (33)	1 **(**33)
Chronic tendonitis, bursitis, or fasciitis	2 (10)	4 (29)	0 (0)	0 **(**0)
Dermatologic				
Skin laxity	1 (6)	6 (40)	3 (100)	0 **(**0)
Wide scars	4 (20)	1 (7)	2 (67)	0 **(**0)
Easy bruising	8 (40)	6 (40)	3 (100)	1 **(**33)
Poor wound healing	1 (5)	0 (0)	1 (33)	0 **(**0)
Cardiac				
Congenital defect*^b^*	5 (26)	1 (7)	0 (0)	0 **(**0)
Chamber enlargement	3 (17)	4 (33)	2 (67)	0 **(**0)
Enlarged aortic root	0 (0)	2 (18)	1 (33)	0 **(**0)
Gastrointestinal disorder*^c^*	1 (5)	5 (33)	2 (67)	0 **(**0)
Hernia or prolapse*^d^*	0 (0)	3 (21)	2 (67)	3 **(**100)

Age of evaluation is shown as mean ± SD; case counts and their prevalence are shown as n (%).

^*a*^Generalized hypermobility defined as a Beighton score of 5 of 9 or greater for children and of 4 of 9 or greater for postpubertal adolescents and adults.

^*b*^Congenital heart defect includes mitral leaflet thickening, structural valve abnormality, aortic stenosis, left ventricular diverticulum, patent foramen ovale.

^*c*^Includes gastroesophageal reflux, irritable bowel syndrome, chronic constipation or stomach pain, and diverticulitis.

^*d*^Includes inguinal, omphalocele, rectal, and umbilical hernia or prolapse.

Although features of hypermobility were seen in each group, the connective tissue dysplasia phenotype was most severe for the CAH-X biallelic individuals, and CAH-X CH-2 was associated with more EDS-type symptomatology than CAH-X CH-1, suggesting the dominant negative effects of the cluster of missense variants in CAH-X CH-2 were more deleterious than the haploinsufficiency caused by CAH-X CH-1 (Table 2). An individual with biallelic CAH-X CH-1/CAH-X CH-3 genotype had the most severe hEDS symptomatology; there was no monoallelic CAH-X CH-3 genotype available to evaluate its standalone significance.

Structural cardiac abnormalities were common among the 39 CAH-X individuals who underwent echocardiogram exams at median age of 13.0 [6.5, 19.5] years, 13 (33%) had at least 1 finding of congenital defect, chamber enlargement or enlarged aortic root, including 7 (36.8%) individuals with a CAH-X CH-1 allele, 4 (28.6%) with a CAH-X CH-2 allele, and 2 (66.7%) with biallelic CAH-X. Cardiac chamber enlargement was particularly prevalent, and was found in 3 (16.7%) individuals with a CAH-X CH-1 allele, 4 (33.3%) individuals with a CAH-X CH-2 allele, and 2 (66.7%) individuals with biallelic CAH-X. There were no structural cardiac defects observed in individuals with *TNXB* splice site mutations.

### Age at Diagnosis

The majority of individuals with classic CAH were diagnosed in early life. As expected, most individuals with a phenotype determined by null (95.3%, Group 0) and In2G (86.9%, Group A) mutations presented in the neonatal period with adrenal crises, atypical genitalia, positive neonatal screening or in utero because of family history. Among Group A, 5.4% of individuals were diagnosed after 1 year of age (range: 3-23.9 years). The greatest variability in age of diagnosis was among those in Group B with 29% of individuals diagnosed in the neonatal period, 12% diagnosed between 4 and 12 weeks of life, 36% diagnosed between 12 weeks and 4 years of age, and 23% diagnosed after 4 years of age, with 1 individual diagnosed after puberty. Overall, 18 individuals in our cohort with prenatal diagnoses were younger siblings of individuals with classic CAH or had a family history of CAH. For individuals in Group BC and Group C, roughly half presented by 12 years of age (Group BC: 56.3%; Group C: 48.4%).

### Biochemical Phenotype at Diagnosis

**Figure 3.**
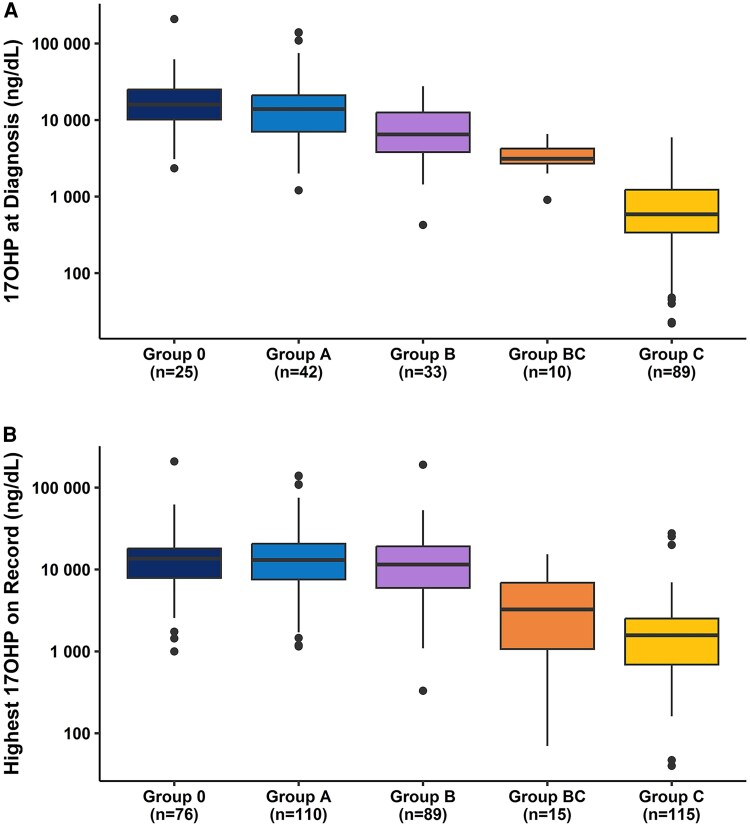
Basal serum 17OHP levels according to 21OHD genotype group. (A) 17OHP at the time of diagnosis and (B) the highest recorded value during clinical care. 17OHP values are plotted on a log10 scale.

Serum levels of 17OHP at the initial diagnosis of CAH were obtained (n = 199). Numbers represent either random 17OHP levels acquired because of clinical suspicion for CAH or baseline values from a cosyntropin stimulation test. 17OHP at diagnosis varied by genotype group (*P* < .001); levels were similar for Group 0 and Group A (median, IQR: 15 909 [10 150-25 056] vs 13 900 ng/dL [7039-21 117] ng/dL; pairwise *P* = .96), but were lower in Group B (median, IQR: 6500 [3820-12 550] ng/dL), Group BC (median, IQR: 3125 [2700-4250] ng/dL), and Group C (median, IQR: 587 [340-1230] ng/dL). Individuals with classic CAH with a genotype expected to result in an SV phenotype (Group B) had lower 17OHP at diagnosis than individuals with classic CAH with genotypes expected to result in an SW phenotype (group 0/A vs B; median, IQR: 14 400 [8255-24 390] vs 6500 [3820-12 550] ng/dL; *P* = .014), although values overlapped and a clear cutoff to establish phenotype was not possible (Fig. 3A).

In contrast to individuals with classic variants, individuals with the NC variants had lower basal 17OHP levels at diagnosis. Moreover, 17OHP levels at the time of diagnosis were higher in Group BC than in Group C (pairwise *P* < .001), with more individuals in Group BC having basal 17OHP levels >1000 ng/dL at diagnosis compared to individuals in Group C (90.0 vs 31.5%; *P* < .001). As expected, all basal 17OHP levels <1000 ng/dL at diagnosis resulted from individuals with NC CAH (n = 63).

### Highest Recorded 17OHP

Compared to 17OHP levels at diagnosis, fewer differences were observed across genotype groupings for the highest recorded 17OHP during clinical care (n = 405) (Fig. 3B). Groups 0 (n = 76), A (n = 110), and B (n = 89) had similar maximum 17OHP values (median, IQR: 13 588 [7885-18 056] vs 13 042 [7572-20 658] vs 11 500 [5972-19 183] ng/dL]; *P* = .44), whereas genotype groups BC and C also had similar maximum 17OHP values recorded (Group BC vs Group C; median, IQR: 3250 [1084-6912] vs 1570 [691-2517] ng/dL, *P* = .61). The maximum 17OHP levels recorded were higher in the classic groups compared to the NC groups (Groups 0/A/B vs Groups BC/C; median, IQR: 12 415 [7255-19 242] vs 1625 [710-2840] ng/dL, *P* < .001). Interestingly, both NC groups had individuals with basal 17OHP levels in the range traditionally associated with classic CAH (>10 000 ng/dL). Within Group BC, 1 individual had a recorded unstimulated 17OHP value of 15 322 ng/dL. In Group C, 3 individuals had unstimulated 17OHP values >15 000 ng/dL (27 647; 25 300; and 19 940 ng/dL).

### Cosyntropin Stimulation Testing in NC CAH

**Figure 4.**
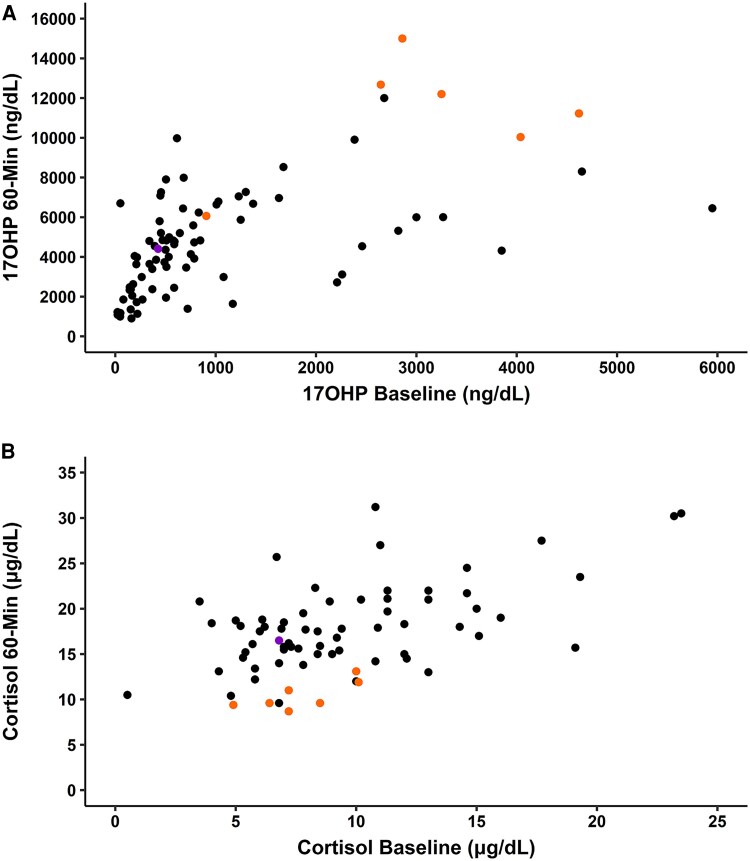
Nomogram of (A) 17OHP and (B) cortisol values at baseline and 60 minutes of a cosyntropin stimulation test in untreated individuals with nonclassic CAH. Genotype Group C is shown in black, Group BC in orange, and Group B in purple.

Data from cosyntropin stimulation testing were available for individuals with NC CAH before starting glucocorticoid therapy (n = 89). At 60 minutes after stimulation, individuals with P30L had higher 17OHP compared to individuals with other NC *CYP21A2* variants (Group BC vs Group C, mean ± SD: 11 201 ± 3011 vs 4458 ± 2357 ng/dL; *P* < .001) (Fig. 4A).

Furthermore, the stimulated cortisol response was significantly lower in NC individuals with the P30L variant compared to individuals with other NC *CYP21A2* variants, most commonly V281L: 60-minute serum cortisol levels increased from baseline on average 2.7 ± 1.3 μg/dL, compared to an increase on average of 8.5 ± 4.3 μ/dL (*P* < .001) (Fig. 4B).

Individuals with cryptic NC CAH were included in this analysis (n = 20). Compared to individuals with symptomatic NC CAH, they had similar baseline 17OHP levels (cryptic NC vs symptomatic NC, median [IQR]: 500 [185-847] vs 590 [369-1675] ng/dL; *P* = .16) but lower values at 60 minutes after cosyntropin stimulation (3310 [1639-4850] vs 4824 [3432-6670] ng/dL; *P* = .018) and no differences were observed in cortisol levels (baseline: 9.2 [6.0-13.7] vs 8.4 [6.8-11.2] μg/dL; *P* = .73; stimulated: 17.9 [15.9-21.0] vs 16.1 [14-19.6] μg/dL, *P* = .24).

## Discussion

This report is the second genetic summary of a Natural History Study conducted at the NIH Clinical Center. Since our 2011 report of a cohort composed of 182 unrelated individuals, our cohort size of individuals with CAH due to 21OHD has grown to 457 individuals from 381 unrelated families. During this time span, our knowledge on 21OHD CAH has deepened thanks to advancements in molecular genetic testing methodologies as well as the increasing availability of comprehensive clinical findings. Accordingly, we have updated our genotyping practices. However, some genotype-phenotype discrepancies remain, albeit at lower rates. Although phenotypic subclassification of the classic forms has fallen out of favor, we describe useful clinical and biochemical differences between various genotype and phenotype groups in individuals who have been diagnosed and followed at the NIH, sometimes for decades.

We have learned that it is important to specify the subtypes of chimeric genes for more accurate genetic diagnosis. Chimeric genes are also termed 30-kb deletions or large/major conversions to reflect the missing chunk of DNA fragment and, in most clinical genetic reports, they are often reported as a classic CAH chimera or a null allele limited to *CYP21A2* (13, 35). Although classic CAH chimeras account for the majority (90.2%) of all chimeras, or an overall 25.9% allele frequency in our cohort, this interpretation ignores the existence of attenuated CAH chimeras and CAH-X chimeras, whose allele frequencies were found to be 1.4% and 4.9% respectively. Because attenuated CAH chimeras feature a *CYP21A1P* promoter *in cis* with a P30L variant, depending on the genetic methodology used, individuals could be misdiagnosed as having either a classic CAH chimera or a P30L allele, leading to inaccurate phenotype expectation of SW or NC, respectively, instead of the expected SV phenotype (19, 32). Moreover, CAH-X chimeras are common, and their overall 10% to 15% prevalence among CAH populations worldwide has been confirmed by multiple large cohorts across different continents (25-27). Despite this, the identification of CAH-X is yet to be included in the scope of most clinical CAH genetic tests, leading to historical underdiagnosis of this condition (13, 36). CAH-X is associated with a broad spectrum of hEDS connective tissue dysplasia manifestations in an autosomal dominant manner and, in our cohort, approximately 33% had associated cardiovascular structural abnormalities. Thus, a genetic diagnosis would be beneficial and allow for proper screening before clinical manifestations occur. We have implemented the specification of chimeric gene subtypes as part of our genotyping practices, which in general has resulted in accurate phenotype prediction related to attenuated CAH chimeras and more comprehensive medical evaluation for patients with CAH-X. We hereby reiterate our appeal of including chimeric gene subtype specification into the standard scope of 21OHD molecular genetic testing (35, 36).

Our large cohort data further demonstrated the overall effectiveness of using a modified version of the CAH genotype subgroup system to estimate/predict phenotypes. This system was particularly accurate in groups 0 and A, which are the most severe CAH genotypes. Notably, 1 individual in Group D with an SV phenotype carried an unspecified 30-kb deletion and a null R356W variant, supporting the importance of subtyping chimeric genes. It is also notable that we did not consider an SV phenotype in group A to be discordant because this is consistently a common finding over time and among various cohorts. Although our overall genotype/phenotype discordance of 3.7% was lower than previously reported large cohorts, we similarly found that discordance occurred mostly in groups B, BC, and C (15, 29, 30). The finding of a more severe phenotype than expected in these genotype groups might be due to intronic variants currently not included in the testing scope, or other elements in the steroid biosynthesis pathway. Specification of attenuated CAH chimeras has significantly improved genetic testing accuracy in genotypes related to P30L. Comprehensive analysis of the promoter region was only performed in 25% of genotypes defined by the standalone P30L variant (Group BC) in our study and is often not evaluated by commercial laboratories, but having an attenuated chimera was excluded. Despite this limitation, overall we found more significant biochemical alterations at diagnosis (higher 17OHP and lower cortisol levels) in genotypes defined by the P30L variant compared to other NC genotypes, supporting the notion that this genotype is indeed intermediate between an NC and SV phenotype and warrants separate consideration. The underlying etiology of this seemingly characteristic broader phenotype is unknown. In addition to genetic or epigenetic factors that equally affect all variants, a posttranslational modulation that is specific/sensitive to the P30L amino acid substitution is possible. Interestingly, our NC cryptic individuals were biochemically similar to symptomatic NC individuals when evaluating basal levels but had lower cosyntropin-stimulated 17OHP, suggesting that lower overall exposure to ACTH-driven adrenal androgens might partially explain their paucity of clinical symptomatology. In general, genotype-phenotype discordance might be caused by broader variation in residual enzyme activity or inaccurate/incomplete genetic testing results, such as misdiagnosis of an attenuated chimera as a null allele, or a missed duplicated functional *CYP21A2* allele. For the genotypes encoding partially functional 21OH, it is also possible that epigenetic factors played a role in altering enzyme abundance, which in turn may have influenced clinical severity as well as hormonal findings.

Levels of 17OHP have been shown to correlate with phenotype and genotype and have been used clinically to distinguish between the various phenotypes (10, 29, 30, 37). We found that the hormonal distinction between SW and SV genotype groups was present at diagnosis, but not later in life, with substantial overlap of 17OHP levels occurring during clinical management, supporting the notion that distinguishing between SW and SV may not be useful in the lifetime management of CAH, especially during adulthood. Alterations in the hypothalamic-pituitary-adrenal axis, adrenal size, or other unknown compensatory mechanisms likely influence the degree of hormonal imbalances observed over time. However, knowing the genotype subgroup and expected phenotype can be valuable in the management of CAH. Several studies have shown that having the SW genotype/phenotype is a risk factor for more severe long-term health outcomes such as worse psychosexual functioning in women (38), impaired reproductive functioning (39, 40), and higher frequency of tumor formation including testicular adrenal rest tumors and myelolipomas (41). Compared to other NC genotypes, the P30L genotype is associated with lower cortisol levels and higher adrenal androgens and may require lifetime glucocorticoid therapy.

Although molecular genetic testing is a vital second tier diagnostic test for CAH, historically, it has been challenging because of the structural complexity of the *RCCX* module. Interference by highly homologous pseudogenes and their commonly existing copy number variations, as typically seen in genome loci of low copy repeats, presents a major obstacle for standard genetic testing platforms such as short-read next-generation sequencing (NGS) and Sanger on conventional PCR amplicons. To date, there are several commercial molecular genetic testing methodologies available in the United States: targeted methodologies such as MMCP and multiplex ligation-dependent probe amplification are low-cost, high-throughput means but their scopes are limited to common variants and their results are often discrepant; some laboratories offer short-read NGS-based 21OHD CAH genetic testing but assure only 90% to 95% accuracy because local genome structural complexity may interfere with the mapping. Sanger sequencing on allele-specific long-range PCR, such as the classic 8.5-kb CYP779f/Tena32f amplicon, offers highly comprehensive and accurate results, and is currently considered the gold standard for 21OHD CAH molecular genetic testing (13). However, it is offered by a limited number of CLIA accredited laboratories due to low throughput and low cost-effectiveness.

A promising advancement in recent years has been the application of long-read based massive parallel sequencing. Compared to standard short-read NGS platforms, long-read sequencing provides contiguous direct sequence spanning tens of kilobases. The newer generation genotyping protocols, currently unavailable in commercial laboratories, permit RCCX module counting and phasing of alleles, exceeding the fragment size and resolution of PCR-based methods (42). As a result, genotypes with compound heterozygous variants can be easily determined without the necessity of parental genotyping (43-46). Some studies also demonstrated that the “mix-up” pseudogene alleles can be recognized or removed by certain software settings so that the difficult allele-specific long-range PCR can be replaced by a relatively easy multiplex PCR. In the future, as sequencing costs continue to decline, whole genome long-read sequencing will likely become the ultimate solution to the challenges caused by the *RCCX* module structural complexity. 21OHD CAH molecular genetic testing based on long-read sequencing is currently not commercially available in the United States. Thus, our practice of implementing both MMCP and Sanger sequencing on allele-specific long-PCR and cross validating by 2 independent CLIA-accredited clinical laboratories was the most comprehensive approach for 21OHD CAH molecular genetic testing.

The limitations of this study are a result of the study design with the participatory nature of subjects and the long span of data collection. The observational natural history study included eligible participant volunteers, and the majority included individuals with classic phenotypes due to recruitment bias over the years to focus our studies on the more severe form of CAH. The retrospective observational nature of this study also included the review of outside medical records to retrieve 17OHP values (at diagnosis and highest value recorded) that inevitably included hormonal measurements using different assays. Importantly, there was no bias in the use of outside medical records across genotypes/phenotypes. Although our subjects came from a more heterogenous genetic background than previously published large cohorts, the distribution of pathogenic variants was similar to prior reports. Lost to follow-up limited the ascertainment of junction sites for a small number of individuals with the common 30-kb deletions. In addition, it is notable that we likely underdetected *CYP21A2* duplication. The methodology of CYP779f/tena32f Sanger sequencing does not test duplication, and we performed duplication testing for a limited number of samples: when genotype-phenotype discordance occurred or duplication was suggested by copy number assessments. Although our approach did not present a complete picture of the *RCCX* module, it did provide accurate and comprehensive genotype information essential for 21OHD CAH molecular genetic diagnosis.

The advantage of a longitudinal natural history study is that the continuity in data collection allows for characterization of phenotypic features over time and enhances our understanding of the evolution of disease progression, and advances in diagnostic techniques and improvements in therapy. Genetically, this has been demonstrated by the evolution of our knowledge on CAH-X and attenuated chimeras over the past several years. Genetic testing remains a vital second tier means to support the primary diagnosis of CAH due to 21OHD based on hormonal and clinical evaluations. Our longitudinal data showed the hormonal distinction between subtypes of CAH, especially classic CAH diminishes with time; however, we also describe important clinical and hormonal distinctions between genotype groups. Implementation of up-to-date knowledge in our genetic testing approach, such as chimera subtype specifications and genotype grouping adjustments to recognize P30L as a unique group, improved its accuracy. Molecular diagnosis of 21OHD is valuable to expand our understanding of the clinical implications of specific genotypes, optimizes genetic counseling, and will ultimately be useful in the clinical management of CAH.

## Data Availability

Some or all datasets generated during and/or analyzed during the current study are not publicly available but are available from the corresponding author on reasonable request.

## Clinical Trial Information

ClinicalTrials.gov identifier: NCT00250159.

## Abbreviations

17OHP: 17-hydroxyprogesterone
21OH: 21-hydroxylase
21OHD: 21-hydroxylase deficiency
CAH: congenital adrenal hyperplasia
hEDS: hypermobility Ehlers-Danlos syndrome
MMCP: multiplex minisequencing and conversion-specific PCR
NC: nonclassic
NGS: next-generation sequencing
NIH: National Institutes of Health
SV: simple virilizing
SW: salt wasting
